# Cause and Manner of Death of a Skeletonized Cadaver: Meeting Some Challenges

**DOI:** 10.7759/cureus.55441

**Published:** 2024-03-03

**Authors:** Ilina Braynova, Verzhiniya Boradzhieva, Pavel Timonov, Antoaneta Fasova, Biliana Mileva, Alexandar Alexandrov

**Affiliations:** 1 Forensic Medicine and Deontology, Medical University Sofia, Sofia, BGR; 2 Forensic Medicine, Alexandrovska Hospital Sofia, Sofia, BGR; 3 Forensic Medicine, University Hospital St. George Plovdiv, Plovdiv, BGR; 4 Forensic Medicine and Deontology, Medical University of Plovdiv, Plovdiv, BGR; 5 Anatomy, Medical University Plovdiv, Plovdiv, BGR

**Keywords:** forensic examination of cadavers, morphological features, gunshot injury, cause and manner of death, decay

## Abstract

Cause of death is defined as a natural disease or injury that led to physiologic changes resulting in death. Manner of death refers to the circumstances surrounding death. Decomposition, especially in advanced stages, creates difficulties in post-mortem examination for it encompasses the processes that lead to the loss of important observable findings and features. Traumatic injuries observed in decomposed cadavers might be analyzed by their vital features and significance for the occurrence of fatal outcomes that help determine the cause and manner of death.

An almost fully skeletonized cadaver was admitted to the Department of Forensic Medicine and Deontology at The Medical University, Sofia, Bulgaria. Along with the obligation to answer the post-mortem interval, what were the anthropological and biological features, the cause and manner of death had to be determined in order to classify the case as criminal or not. The cause of death was established by the morphological finding - gunshot injury of the head, passing the brain. The manner of death remained undetermined because of the absence of soft tissues in the areas of the injuries. It was concluded that there was no sufficient forensic data to answer if it was suicide, homicide, or even an accident.

## Introduction

Cause of death is defined as a natural disease or injury that led to physiologic changes resulting in death [1]. Manner of death refers to the circumstances surrounding death [2]. Forensic autopsy is crucial for the correct determination of the cause and manner of death [3]. Medicolegal or forensic autopsies are performed in order to provide information about the identity, cause of death, postmortem interval, and circumstances of death. This is essential for investigation when they have to solve a possible crime [4]. In most jurisdictions, the manner of death is classified into five major categories:

1. Natural: due to natural disease processes.

2. Homicidal: due to a violent and criminal act of another person with the intent to cause fear, harm, or death and some negligent acts, even when a person did not intend to cause harm.

3. Suicidal: due to fatal self-inflicted injury or intoxication.

4. Accidental: due to injury when there is no evidence of intent to harm.

5. Undetermined: in cases with insufficient information regarding the circumstances of death to determine manner [5].

Decomposition might be the reason for various difficulties in postmortem examination for it encompasses the processes of autolysis, putrefaction, and decay that lead to destruction and loss of tissues [6]. The changes in cadavers during decomposition make all of the aspects of their forensic analysis a complex problem [7]. Various intrinsic and extrinsic factors impact the process of decomposition and putrefaction. Some of the intrinsic factors such as cause, and manner of death have an important role that affects the process of decay. On the other hand, the putrefaction itself affects the appearance of the morphological findings that are used in the determination of cause and manner of death [8]. Postmortem decomposition of the cadaver can mask or disrupt the classical features of a skin lesion, creating difficulties in the establishment of the cause and manner of death. When the skin and soft tissues are fully decomposed and missing some of the characteristic features of the injuries are inevitably lost [9].

Decomposed bodies are often recovered from open areas. Even though some of the informative features are lost due to decay it is not correct to regard the autopsy of a decomposed body as unrewarding [10]. The recovery of severely altered cadavers such as decomposed ones can be challenging for forensic examiners due to the difficulties in identification, postmortem interval estimation, and cause and manner of death determination [10]. When present, traumatic injuries in decomposed cadavers might be analyzed by their vital features and significance for the occurrence of fatal outcomes that help determination of the cause and manner of death [11].

## Case presentation

An almost fully skeletonized cadaver was admitted to the Department of Forensic Medicine and Deontology at The Medical University, Sofia, Bulgaria. The forensic examination of the human remains was ordered by the police. The forensic examiners had to answer to the following questions:

1. Are the remains human in origin?

2. What was the age of the deceased?

3. What was the biological gender of the deceased?

4. Were there any observable traumatic injuries?

5. What was the cause of death?

6. Is it possible, in case of establishment of traumatic injuries, the latter to be self-inflicted or not (What is the manner of death?)?

7. What was the postmortem interval?

**Figure 1:**
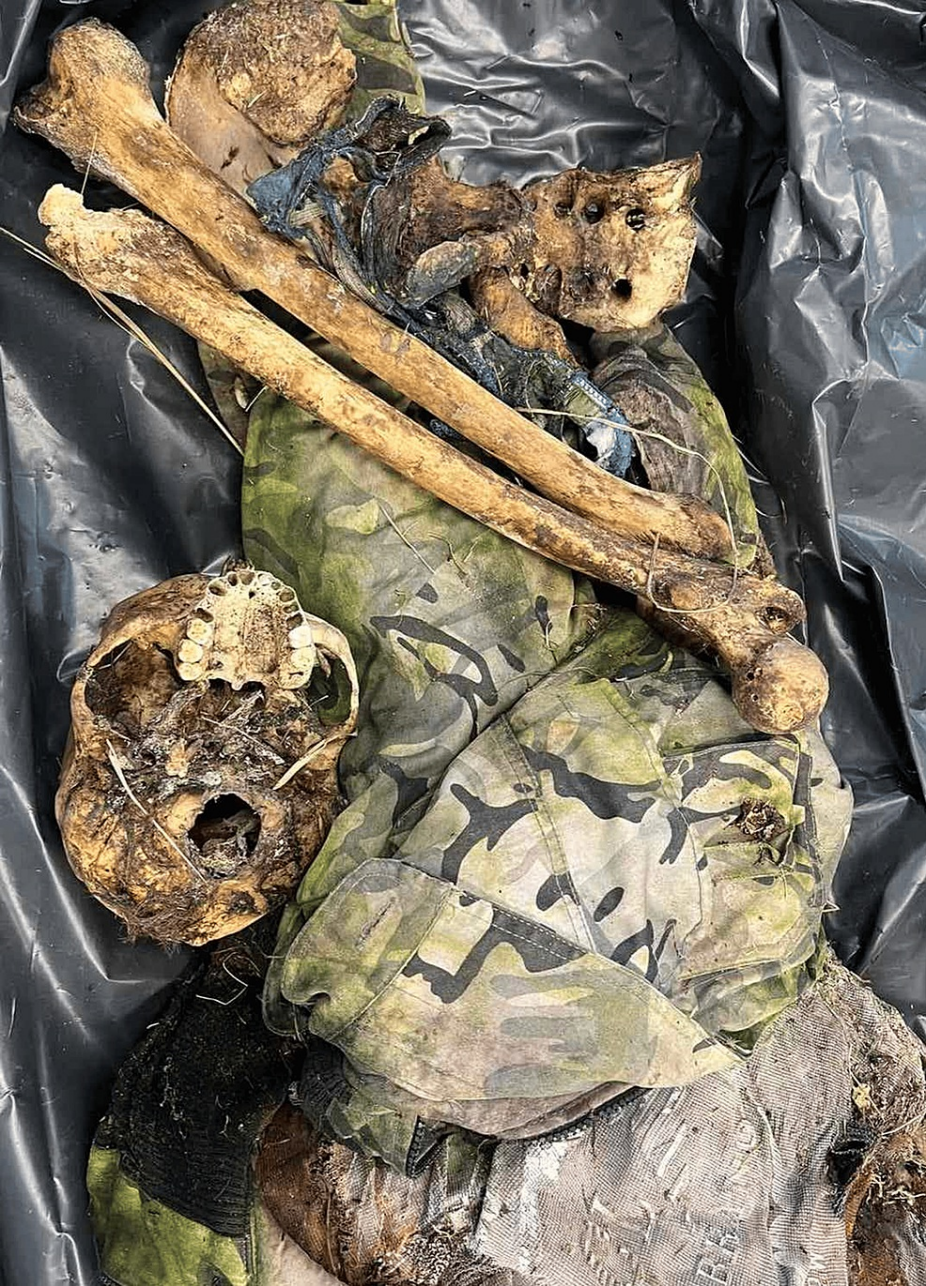
General view of the cadaver. There are visible clothing remnants and mostly skeletonized bones and remnants of soft tissues.

In order to give answers to the aforementioned questions, and any other relevant questions, the forensic examiners had to perform a thorough examination of the cadaver remnants. In the beginning, the forensic examiners got acquainted with the information collected by the investigators who attended the death scene. The cadaver was found in a wood by a hunter’s dog that brought a bone to the hunter. He saw the almost fully skeletonized body and called the police. They noted hole-like fractures in the skull and found a rusty pistol right next to the decomposed body. There were clothes over the cadaver. The cadaver was transported to the forensic department for further examination. The examination in the department started with the static observation of the clothing and visible parts of the cadaver (Figure 1), which all were in a zipped black bag.

The clothing contained a jacket, with a military stamp, a T-shirt, and other remnants that could not be identified as certain clothes. The cadaver itself was almost fully decomposed and skeletonized. Only dry remnants of soft tissues were present. There were also hair remnants, stuck on the skull. Some of the articulations were destroyed too. The bones were heavy and still greasy.

**Figure 2:**
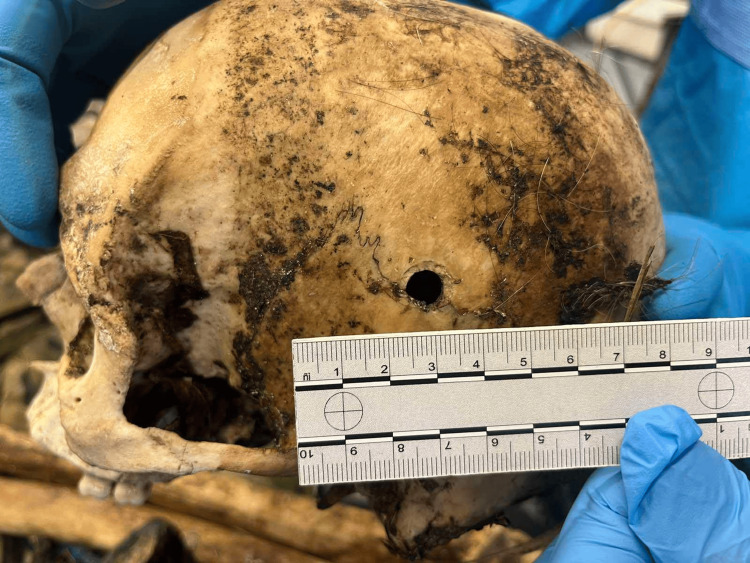
Entrance wound – outer view. The entrance is in the left posterior temporal area, approximately 0.9-1cm. It is roundish and almost regular with typical morphology of gunshot entrance.

The bones of the head were present with the exception of the mandible that was missing. The bones were massive, with expressed roughness and bony ridges, the orbits were square-like - features, more characteristic for the male gender. In the back border of the left temporal-parietal area of the skull, there was a hole-like fracture, with a round shape, approximately 0.9-1cm in diameter (Figure 2).

**Figure 3:**
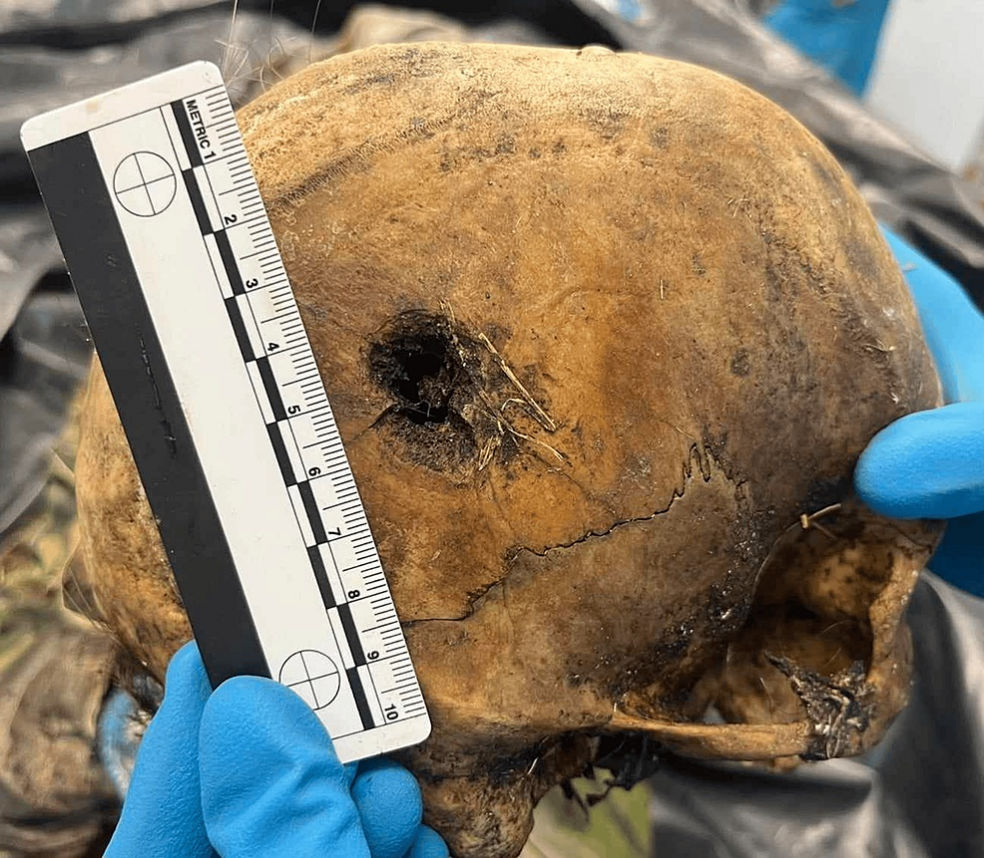
Exit wound – outer view, external beveling. The exit is in the right posterior temporal area, approximately 0.9-1cm of the fracture itself. There is observable external beveling with typical morphology of gunshot exit.

On the right side of the skull, there was another oval fracture, corresponding to the first one with approximately the same diameter (Figure 3).

**Figure 4:**
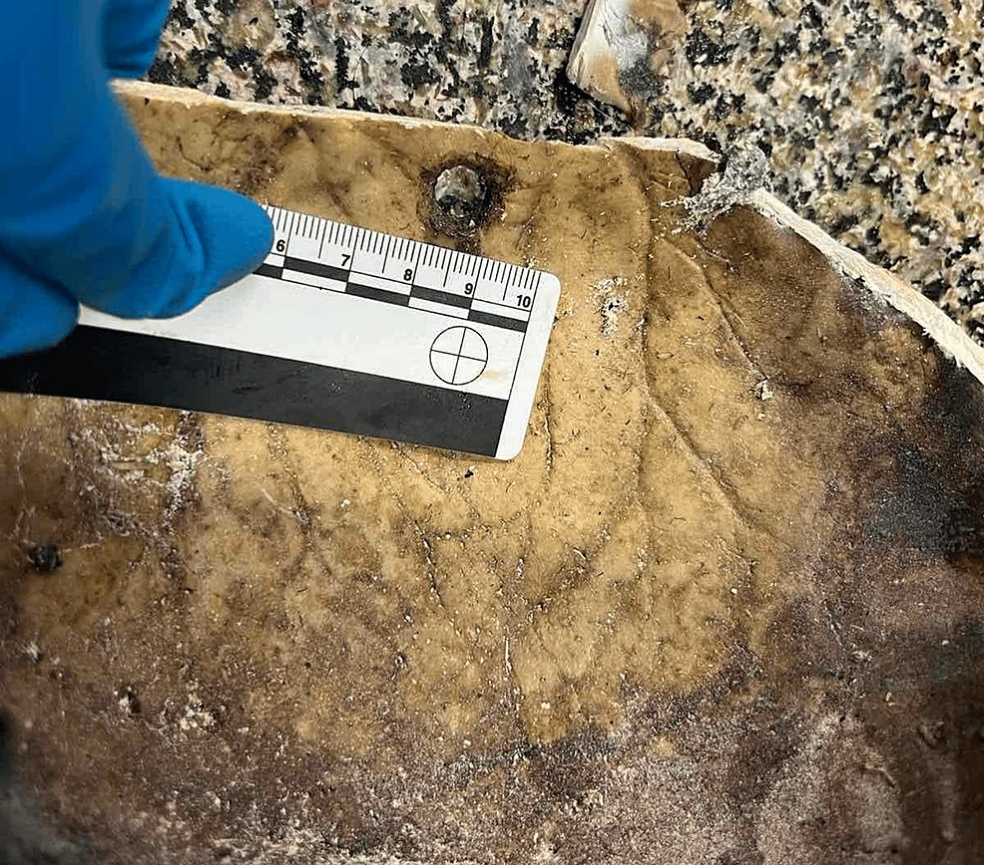
Entrance wound – internal beveling.

**Figure 5:**
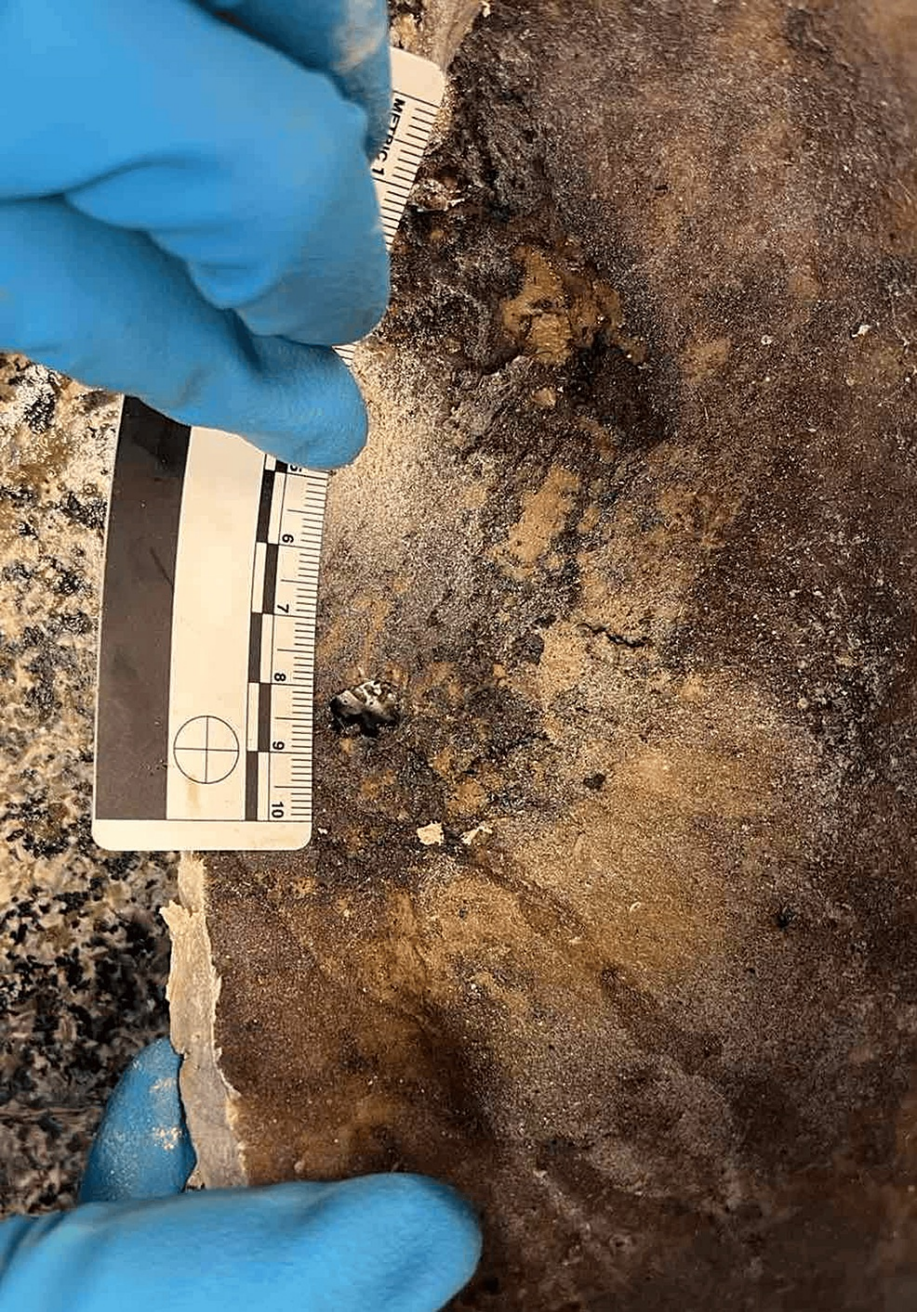
Exit wound – internal view.

Both of them had the shape of double-crossed cones, with smaller diameter on the external bony plate for the left fracture and for the right fracture - on the internal bony plate. In other words, there was obvious beveling - internal for the wound on the left side (Figure 4) and external for the injury on the right side (Figure 5).

**Figure 6:**
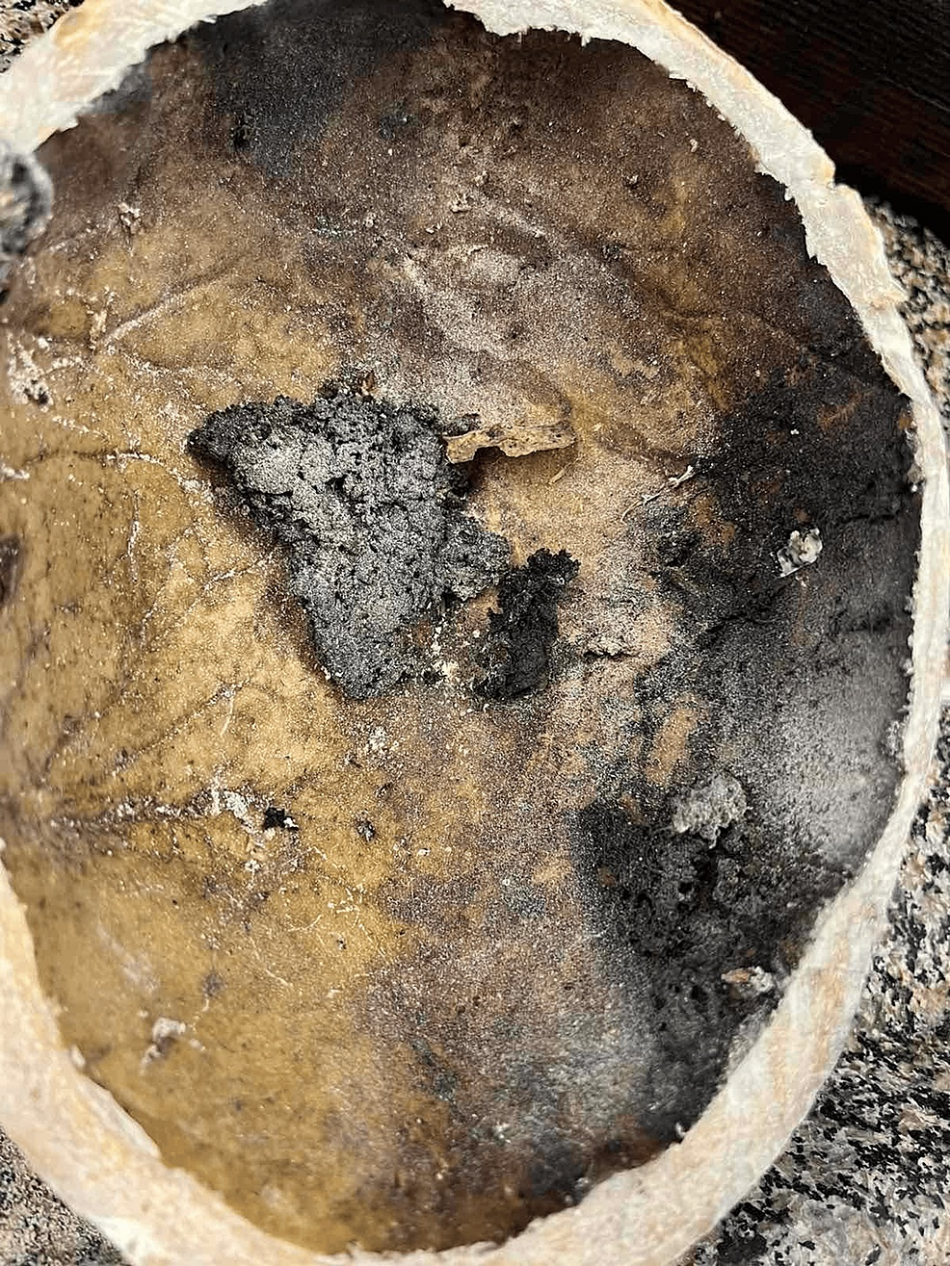
Dark reddish-brown decomposed substance – most probably remnants of intracranial bleeding.

They were connected by a probe in a wound channel. By their appearance, location, and morphological features, they were determined to be part of the wound channel of a fire-arm injury. The direction of the injury in the skull was from the left side to the right side. After the skull was opened with electrical saw, revealing the internal morphology of the injuries in the bones, there was another interesting finding. There was almost no content into it, instead of dark reddish-brown, almost black, substance in the right temporal and parietal areas, sticking tight to the internal bony plate of the skull (Figure 6). It looked like remnant of decomposed blood collection located in these areas.

Three molars were present in the upper jaw (7,6|6). The other teeth were missing. The present teeth were without specific features, without significant pathological changes and erosion. 

The other part of the cadaver contained cervical and thoracic vertebrae, ribs, long bones of the left upper extremity, the femoral bones and the pelvic bones. The length of the femoral bones was measured. The pelvic bones and the whole pelvis had masculine configuration. 

Based on the full examination of the remnants, the conclusions were that they were of human origin, the gender was male. This conclusion was based on the observable anthropological features, mainly the shape and the features of the skull, the shape of the orbital bones, and also the shape of pelvic bones and the whole pelvis. The approximate age interval was between 30 and 50 years old. The postmortem interval most probably was between one and two years.

The most considerable to the investigator's questions - about the cause and manner of death, could be only partly answered. By the present findings it could be concluded that it is highly possible that the cause of death is gunshot injury of the head. There were no other observable injuries in the cadaver remnants. Because of the absence of soft tissues there was no material for additional forensic and ballistic examinations that could determine the distance of shooting. Based only on the morphology and localization of the injury, no satisfactory conclusion could be drawn about the manner of death - in could be either suicidal, or homicidal. Accidental manner could not be also excluded. The difficulties in manner of death determination reflected some of the inevitable limitations of forensic examination of decomposed cadavers that might lead to drawing of insufficient and unsatisfactory conclusions.

## Discussion

In the present case, two of the major expert problems that had to be solved were the determination of the cause of death and the manner of death. The other conclusions concerning the postmortem interval, the age, gender, height, and other biological features of the deceased were also important for the investigation. Nevertheless, the latter were mostly concerned with revealing the cause, and especially the manner of death, or, in other words, if there was or there was not a crime.

The cause of death was not so difficult to establish, having the morphological finding showing gunshot injury of the head. It obviously was passing the brain and severe enough to cause the fatal outcome. The direction of the wound channel was established based on the typical in this case morphology of the injuries - oval entrance wound (with internal beveling that is observed in more than 90% of the entrances of the skull [12]) on the left side and beveled exit injury (with external beveling) [13] on the right side. External beveling is observed in two-thirds of the exit injuries in bones, while internal beveling is not noted in exit injuries [14]. The possibility of postmortem injuries imitating gunshot wounds [15] was also discussed, but there were no arguments supporting such suggestions.

A problem that was more complex, and even impossible to solve was the manner of death. The most probable alternatives were homicide or suicide. Based on the findings of the present tissues that were examined, both of them were equally possible and this fact was pretty disturbing for the investigation and too unsatisfactory for the forensic examination and conclusions. The loss of soft tissues, including skin, was the main obstacle to a more profound analysis and determination of the distance of shooting that could help determination of the manner of death. There are some practical reports of detection of gunshot residue in larvae that could be diagnostic in decayed cadavers [10]. In our case there were no larvae, because of the long postmortem interval (approximately between one and two years since the death), so, unfortunately, this method could not be used.

## Conclusions

There are various difficulties in the examination resulting from the putrefaction of cadavers. This does not mean that all of the conclusions would be unsatisfactory. There is practically no state of decomposition that should stop a forensic doctor from examining human remnants. Depending on the case, the investigation prioritizes based on the specific importance of some of the conclusions. In the present case, the death was violent, and this was a basic key point for the investigation. That is why it was important for the police to report most accurately what were the cause and manner of death. The biological and anthropological features were essential too. The forensic examiners met no actual difficulties establishing the cause of death observing the typical injuries of the skull that positively were due to gunshot in the head. The difficulties came when the manner of death had to be discussed. Both suicide and homicide were equally possible. The findings - advanced stage of decay of the body, and loss of soft tissues, especially the skin, were not sufficient to draw a certain objective conclusion about which one of those alternatives was the actual manner of death. In such cases when there are limitations of the forensic conclusions, it is up to the investigation to decide what exact manner of violent death is the present case.
